# Ankle/brachial index to everyone

**DOI:** 10.1186/1471-2482-12-S1-S18

**Published:** 2012-11-15

**Authors:** Giuseppe Giugliano, Anna Sannino, Linda Brevetti, Cinzia Perrino, Gabriele Giacomo Schiattarella, Anna Franzone, Federica Serino, Marco Ferrone, Fernando Scudiero, Andreina Carbone, Michele De Paulis, Raffaele Izzo, Bruno Amato, Bruno Trimarco, Giovanni Esposito

**Affiliations:** 1 Department of Clinical Medicine and Cardiovascular and Immunological Sciences, “Federico II” University”, via Pansini 5, 80131, Naples, Italy; 2 Department of General, Geriatric, Oncologic Surgery and Advanced Technologies, “Federico II” University”, via Pansini 5, 80131, Naples, Italy

## Abstract

**Background:**

In the last years significant attention has been paid in identifying markers of subclinical atherosclerosis or of increased cardiovascular risk.

**Method:**

An abnormal ankle/brachial index (ABI) identifies patients affected by lower extremity peripheral arterial disease, and even more important, represents a powerful predictor of the development of future ischemic cardiovascular events.

**Conclusions:**

In our opinion, ABI is a cardiovascular risk prediction tool with very desirable properties that might become a routine measurement in clinical practice.

## 

Cardiovascular diseases are the leading causes of morbidity and mortality in the western world [1-4], and atherosclerosis is the underlying cause of the majority of cardiovascular diseases [5,6]. Lower extremity peripheral arterial disease (LE-PAD) is one of the main manifestations of atherosclerosis affecting about 20% of the population aged 55 and older [7,8]. It has been calculated that about 27 millions people in Europe and United States suffer from this pathology [9], thus representing a socio-economic problem of great magnitude [10]. In particular, elderly populations have more severe forms of atherosclerosis with a higher prevalence of polidistrectual disease including carotid arteries and abdominal aorta [11-13] and develop a higher grade of disability compared with younger people [14]. In addition to be an important cause of disability in its symptomatic forms (intermittent claudication and critical limb ischemia), LE-PAD is associated with an elevated risk of developing ischemic cardiovascular events [3,6,15-17], which is similar in symptomatic and asymptomatic patients [16-20].

LE-PAD diagnosis can be made simply, accurately and non-invasively by ankle/brachial index (ABI) measurement [8,18,21]. The assessment involves placing a sphygmomanometer cuff just above the ankle and using a Doppler instrument to measure the systolic pressure of the posterior tibial and dorsalis pedis arteries of each leg. The ABI is then obtained dividing the systolic pressure of each of the ankles by the highest brachial pressure of either arm [21]. A patient’s ABI is defined as the lowest of the leg ABI measurements (Figure 1). A resting ABI value ≤0.90 defines the presence of LE-PAD and it has a sensibility of about 95% in identifying the presence of a hemodynamically significant arterial stenosis at angiography between heart and foot and near 100% specificity in excluding a normal subject [18]. Furthermore, ABI gives important information about LE-PAD severity, which is higher with a lower ABI value. In addition to its diagnostic utility, an abnormal ABI value represents a powerful predictor of the development of future ischemic cardiovascular events [3,18,21,22]. Such risk increases with the decrement of the ABI value and it is independent of the presence or absence of the classic cardiovascular risk factors [22]. In this regard, it is important to note that although classic cardiovascular risk factors are useful to predict risk in populations, their accuracy in predicting cardiovascular risk in individuals varies considerably. Indeed, in the last years significant attention has been paid in identifying markers of subclinical atherosclerosis or of increased cardiovascular risk in humans as well as animal models [23-27]. Although several tools have been proposed [27-29], frequently the clinical utility of measuring such markers remains uncertain for several reasons, including costs, low reproducibility, conflicting studies or lack of confirmatory studies, and lack of measurement standardization [27]. Additional research will be needed to quantify the impact and cost effectiveness of these markers on patient management and clinical outcomes, with simplicity of assessment and low cost being the essential characteristics of an optimal risk marker.

**Figure 1:**
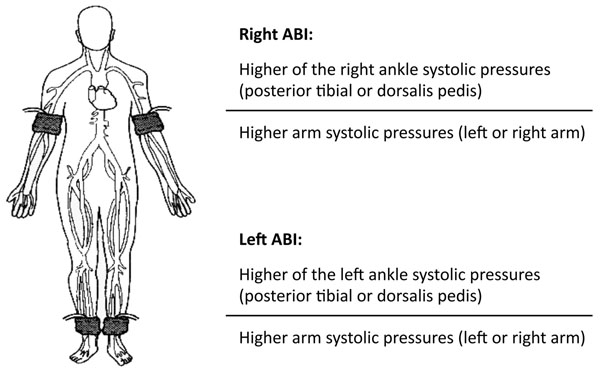
How to perform ankle/brachial index.

Given that the vast majority of LE-PAD patients is asymptomatic and that an abnormal ABI has an important power in predicting the occurrence of future cardiac and cerebrovascular ischemic events, it would be extremely important to identify which populations need to be screened with this inexpensive tool. Several epidemiologic studies, including the PARTNERS [8], and the latest guidelines [18,21] suggest that subjects who should undergo to ABI measurement are:

• All subjects who have exertion leg symptoms (also atypical) or not healing wounds;

• All subjects of 50 years and older with a history of smoking or diabetes;

• All subjects with age >65 years independent from the presence of cardiovascular risk factors.

Although such recommendations are already quite extensive, in our opinion they could be even broadened, given the non-invasive nature, the low cost and the elevated sensibility and specificity of ABI. In particular, all patients with coronary artery disease (CAD) should be screened, since in this population the presence of LE-PAD is relatively frequent (16-20%), entails a higher severity of the coronary disease [30,31], and is associated with a worse prognosis [32]. Given the multidistrectual nature of atherosclerosis, also patients affected by cerebrovascular disease should undergo ABI measurement to identify, like in CAD, a subgroup at even higher risk that should receive higher clinical consideration.

Noteworthy, ABI has the power to provide additional risk stratification of those subjects who have a 10-year intermediate cardiovascular risk (between 10 and 20%) [21,33]. In these individuals, the finding of an abnormal ABI value switches the patients towards a higher cardiovascular risk which needs secondary prevention, while a normal ABI could lower the risk estimation to the need of primary prevention. Identification of individuals with asymptomatic lower extremity PAD is of utmost importance, so that therapeutic interventions known to diminish their increased risk of myocardial infarction, stroke, and death may be offered [3]. Smoking cessation, lipid lowering, diabetes and hypertension treatment and antiplatelet therapy are recommended to reduce the risk of adverse cardiovascular ischemic events [34]. In this context, it should be acknowledged that statins are particularly effective in reducing cardiovascular risk of patients with LE-PAD, even if with normal cholesterol levels [35]. In conclusion, because of the simplicity of execution and for the diagnostic and prognostic importance, ABI, in our opinion, might become a routine measurement in clinical practice, also in the general practitioner setting. Indeed, early identification of subclinical atherosclerosis and LE-PAD might offer a unique opportunity to put on time into effect the necessary prevention measures.

## List of abbreviations

LE-PAD: Lower Extremity Peripheral Arterial Disease; ABI: Ankle/Brachial Index; PARTNERS: PAD Awareness, Risk, and Treatment New Resources for Survival; CAD: Coronary Artery Disease.

## Competing interests

The authors declare that they have no competing interests.

## Authors’ contributions

GG: conception and design, interpetration of data, given final approval of the version to be published; AS, LB, CP, GGS, AF, FS, MF, FS, AC, MDP: acquisition of data, drafting the manuscript, given final approval of the version to be published; RI, BA, BT: critical revision, interpretation of data, given final approval of the version to be published; GE: conception and design, critical revision, given final approval of the version to be published.
